# Social Networks in Limbo. The Experiences of Older Adults During COVID-19 in Ghana

**DOI:** 10.3389/fpubh.2021.772933

**Published:** 2021-11-17

**Authors:** Emmanuel Akwasi Asante, Kofi Awuviry-Newton, Kwamina Abekah-Carter

**Affiliations:** ^1^Institute of African Studies, College of Humanities, University of Ghana, Accra, Ghana; ^2^African Health and Aging Research Center (AHARC), Winneba, Ghana; ^3^Department of Social Work, University of Ghana, Accra, Ghana

**Keywords:** social network, COVID-19, lived experiences, loneliness, quality of life, Ghana

## Abstract

While studies exploring COVID-19 and its global influence have begun, social networks and support among older adults in low-and middle-income countries, such as Ghana have been inadequate despite its enormous relevance. Thus, the study presents the voices of older adults in Jamestown, Accra and their social networks during the COVID-19 pandemic in Ghana. Using a phenomenological approach, data were collected from 15 older adults through in-depth interviews on older adults' social network experiences during COVID-19 pandemic situation. Older adults generally struggled to maintain connections with their family members, friends, neighbors, and the community, especially during the lockdown. They ascribed their limited interaction to COVID-19 preventive measures, such as social distancing and the limitation of face-to-face meetings imposed by the government. Loneliness, stress, and depression are also linked to the breakdown of social networks. The findings provide a deeper understanding of the impact of COVID-19 on older adults' quality of life. It emerged that the Ghanaian society could reconsider the professional services of gerontologists, social workers, community outreach workers, and philanthropists in mitigating loneliness, stress, and depression among older adults in current and future pandemics.

## Introduction

Before the COVID-19 pandemic, social network support was identified as a critical element in older adults' wellbeing. According to Kaniasty (1), social support and social connectedness have substantial immediate effects on older adults' health and wellbeing during natural disasters. However, the outbreak of the COVID-19 pandemic and the non-pharmaceutical preventive measures, such as social distancing, limited face-to-face contacts, staying at home (2), as well as the exposure of vulnerable groups, like older adults have critically altered the operations of relationships worldwide. Evidence suggests that restrictions and regulations to prevent the rapid spread of infection have been tightened for older adults, resulting in a significant shift in their social relations (3). For instance, a large population study of older adults conducted in the United States of America and Denmark (1,013) found that older adults faced emotional loneliness after the stay-at-home orders were implemented (4, 5).

In an attempt to understand the state of social networks, it is necessary to highlight the different non-pharmaceutical measures and their impact on social networks. As indicated earlier, these COVID-19 precautionary measures include social distancing, staying at home, reduction in face-to-face interaction including the lack of physical contact with friends and neighbors, as well as the ban on social activities. These restrictions may pose multiple risks of stress, loneliness, stigma, and trauma-informed PTSD to all persons including older adults (2). Additionally, they seem to have worsened previously existing social networks with families, friends, and neighbors, which serve as a social support system for older adults. In extreme cases, these measures may be difficult for older adults to cope with since their physical and mental health is heavily reliant on their social interaction. Without doubt, WHO—imposed restrictions by the various governments placed many older adults' daily life activities and social networks in limbo.

Multiple studies have revealed that older adults with strong social networks have better mental health (6–8). Significantly, social networks serve as a substantial source of personal happiness (9). However, the growing burden of infectious diseases, such as COVID-19 has begun to have a negative impact on the importance of social networks for the wellbeing of older adults. Older adults with one or two comorbidities have a higher risk of being infected with COVID-19 and experiencing episodes of mortality (10–12). A preliminary study conducted on COVID-19 patients in Ghana showed that people with the most common underlying illnesses, such as hypertension, diabetes, asthma, heart disease, and chronic kidney disease were diagnosed with COVID-19 (13). This places older adults, particularly those suffering from diabetes and hypertension, at a higher risk for severe illness from COVID-19 (14), resulting in an increase in stress, depression, and isolation during the peak of COVID-19 (2).

It is reported that most older adults are unable to carry out daily tasks without assistance (15, 16). In Ghana, for example, older adults are treated with dignity and respect, and they require assistance to carry out their daily activities (15, 17). The support provided by relatives or close kin, marriage partners, friends, and social capital are considered as social networks, enabling many older adults to remain in their homes (18), considering their need for long-term care (15). Among these networks, kin or family ties play a central role in older adults' wellbeing in Ghana (19). It is perceived that older adults who interact with each other more and their support networks frequently appear to have fewer depressive symptoms (20). But in recent times, growing old, on the other hand, has become a burden in Ghana because the availability of caregivers is uncertain (15).

While research on COVID-19 (21–23) has begun to examine social support networks among older adults living in developed countries, a few studies (24–26) have examined these issues within the context of Ghana. Thus, the purpose of this study was to explore the experiences of older adults and the impact of COVID-19 on their social networks and support systems during the peak of COVID-19.

## Materials and Methods

In this study, we employed a qualitative phenomenological approach to explore the experiences of older adults regarding their social networks in the ongoing COVID-19 pandemic. Phenomenology was selected because it focuses on older adults' perceptions and experiences and involves thoughtful planning and skillful interviewing (27). This approach is appealing for this study because it helps to explore the feelings, experiences, as well as health problems from the perspective of older adults in the context under study (28). Furthermore, the approach requires researchers to collect data, analyze it, and report on the findings of individuals' lived experiences (29). Therefore, the role of the researchers was to understand, interpret and report on the state of social networks among older adults and how COVID-19 has complicated such bonds.

### Study Setting

Jamestown covers a total land area of approximately 30 km^2^, and it encompasses vicinities, such as Bukom, Usshertown, Adedainkpo, Sawalaba, Korle Woko, and Akoto Lante. According to the 2010 Population and Housing Census, while the total population of the Accra Metropolis was 1,665, 086 (females 51.9%, males 48.1%), representing 3.1% of the total population of the Greater Accra region, the elderly population was estimated at 65, 920, including Jamestown (30). Majority of the inhabitants of the Jamestown community are *Gas*. Jamestown is home to many fishermen, merchants, boxers, footballers, and artists. It is also close to the central business area of Greater Accra region. The study area was chosen because it was marked as one of the hotspots of COVID-19 infectious diseases in the Greater Accra region.

### Recruitment Procedure

This study was conducted on 15 older adults consisting of 10 females and five males living in the Jamestown community in the Accra Metropolis. Evidently, this sample size resulted in a considerable gender imbalance. A purposeful sampling method was used to recruit the research participants. The use of this sampling technique allowed the researchers to recruit participants based on a set criterion. Specifically, participants in the study (aged 60 and over) included residents of Jamestown in Accra, had lived in the community for at least 45 years, and could speak and hear conversations without any assistance. Since older adults who are seriously ill may be at risk of COVID-19 infections during physical contact, their health status was also considered during the selection process. To reach the participants, the first author, who had stayed in the study area for years, proceeded to visiting the homes of older adults to identify potential participants, brief them about the study and its aim, and seek their consent to take part in the study. Out of about 25 older adults who were contacted throughout this exercise, only 15 of them showed interest to participate in the research.

### Data Collection

Data were collected through in-depth interviews to explore the participants' experiences with COVID-19 and its impact on their social networks. Interviews lasted for an average of 60 min and were conducted in April 2021 during the second wave of COVID-19 in Ghana. In light of the fact that older adults are more susceptible to COVID-19, the data collection period chosen for this study was timely and appropriate because the spread of COVID-19 had slowed and interaction with people had become easier, minimizing the risk of COVID-19 infection in older adults. The time frame for data gathering, on the other hand, did not compromise the study in any manner. We employed semi-structured techniques to collect data from the participants. Participants were asked questions pertaining to their experiences during the lockdown and highly restricted orders imposed by governments during the first wave of COVID-19. Regarding the interview questions, participants were asked about their relationships with family members, friends, and associations and how COVID-19 has negatively impacted these networks. These questions were posed to determine the state of social networks among older adults during the COVID-19 pandemic peak. More importantly was how older adults dealt with, and continue to deal with, loneliness, stress, and stigma associated with COVID-19. Through our engagement, we were able to build a rapport with the participants, removing potential difficulties between the researchers and the participants. Participants were able to speak openly about their social networks and the effects of COVID-19 restrictions on their support networks.

Before each interview session, participants, as well as their available family members and caregivers were informed about the specific objective of the study, as well as the participant's right to anonymity, confidentiality, and the right to withdraw from the study at any time they desired. The first author conducted the interviews *via* the languages they were more proficient in (English and Twi). Nonetheless, in view of the fact the study area is known for speaking the Ga language, the researchers adopted a strategy to engage participants who understood the content of the interview questions but struggled to articulate themselves in any of the two languages. Thus, in cases where participants understood none of the languages, interpretation was done by members of the participant's household or caretakers, who understood either Twi or English. During each interview session, measures were also put in place for each participant to wear a nose mask and sanitize their hands. Interviews were audio recorded.

The data reported in this paper is part of larger ongoing research which is exploring people's (including older) perceptions, beliefs, and attitudes about the COVID-19 pandemic.

### Ethical Considerations

Observing ethical guidelines, the consent of the participants was taken before the interviews, and their identities remained anonymous. The study acquired a formal ethical clearance certificate (ref: CHRPE/AP/108/21) from the Committee on Human Research, Publication, and Ethics (CHRPE) of the Kwame Nkrumah University of Science and Technology, Kumasi. The study followed the Ethical and Protocols Review procedures of CHRPE and administered to all the study participants. The signed consent included an introductory letter from the Institute of African Studies at the University of Ghana, which was used to introduce the researchers to the study participants.

### Analysis

The thematic analysis as a qualitative inquiry was employed to analyze the experience of older adults regarding the social networks during the COVID-19 pandemic. Thematic analysis was chosen because it is often used in qualitative descriptive or phenomenological approaches of qualitative designs (31). It also highlights the social, cultural, and structural factors that shape individual experiences, allowing for the construction of knowledge through interactions between the researcher and research participants, revealing socially and culturally constructed meanings (31, 32). Furthermore, thematic analysis is a systematic approach to data analysis that gives each data item equal attention and identifies important parts of the data. Transcribed data was reviewed and identified relevant themes for the study objective, as well as well as produce a detailed explanation of each subject. The transcripts and audios were double-checked to ensure data quality to verify that they accurately represented the information provided after the interviews. Regarding the credibility and reliability of the study, participants' views were presented in the form of quotations to give more insights. In addition, the relationship between themes and interview questions was examined, which aided in the validation of data and the accurate reporting of key themes revealed by participants. Furthermore, one of the co-authors independently evaluated the transcripts, bracketing data based on preconceived notions and rigorously following the phenomenological strategy. The findings were then forwarded to the other two authors, who collaborated and cross-checked, discussed, and agreed on themes (33).

## Results

Participants were from the Jamestown community, and they included 10 females and five males. The data is organized according to the primary themes that emerged from the analysis. We also examined and presented the findings based on the themes that emerged during the interviews. These are “*participants' perception of COVID-19*,” “*relationships with people before and during COVID-19*,” and “*depression, and loneliness*.”

### Sample Demographics

Participants who took part in the study were between the ages of 60 and 80 years. See Table 1 for details of the characteristics of the study participants.

**Table 1:** Demographic information of participants.

**Pseudonyms**	**Gender**	**Age**	**Living arrangement**	**Marital status**	**Education**	**Employment**
1st	Female	70	Live with children and husband	Married	Elementary	On pension
2nd	Female	77	Live alone	Widowed	None	Not working
3rd	Male	67	Live with children and wife	Married	None	Seller
4th	Female	75	Live alone	Widowed	None	Not working
5th	Male	64	Live with children and wife	Married	None	Seller
6th	Female	67	Live with children and husband	Married	None	Trader
7th	Male	73	Live alone	Widowed	None	Not working
8th	Male	71	Live alone	Widowed	None	Not working
9th	Female	69	Live with children and husband	Married	Elementary	On pension
10th	Female	69	Living with children and husband	Married	None	Trader
11th	Female	78	Live alone	Widowed	None	Not working
12th	Female	70	Living with children and husband	Married	None	Not working
13th	Male	72	Live alone	divorced	None	Not working
14th	Female	66	Live with children and husband	Married	None	Seller
15th	Female	60	Living with children and husband	Married	None	Seller

### Perception About COVID-19 Pandemic

Some older adults perceived the COVID-19 pandemic as untrue and a ruse. Almost all the participants explained that since the outbreak of COVID-19, they have not seen or met anyone who tested positive. As a result, they find it difficult to believe whether COVID-19 exists or not. Five participants narrated that the COVID-19 pandemic is just a name but does not exist. They believe that Europeans or white people created the name to frighten Africans to steal their resources. However, some of the participants admitted that COVID-19 is real and is killing Jamestown people. Some participants, on the other hand, did not believe in the existence of the virus:

*I do not believe that COVID-19 exists in Ghana. Even if it does, it cannot be found in this famous community. Sometimes we are made to drink seawater, which prevents people from contracting diseases, not to mention death. When someone gets wounded, they are treated with seawater first before going to the hospital. Of course, I have been told people are dying from COVID-19 but I have not seen an infected person in this community (78-year-old-woman)*.

*In Africa, people die of malaria. COVID-19 is said to be caused by a virus and the symptoms are similar to malaria. Malaria killed my friend some years back, and I fear malaria more than any other disease. But I did not believe COVID-19 was real until I saw someone struggling to breadth. I am told that people like us, especially older adults with weak immune systems, are susceptible to the virus. My point is that if it is true that COVID-19 is real, then we should all have died by now. So COVID-19 is not true, but malaria is (70-year-old-woman)*.

Participant 10, a 69-year-old female, admitted that, even though she has not seen any infected person or tested positive, she believes the virus is real. She commented that many people in the community do not believe the COVID-19 pandemic is true. She revealed that she had tried to advise and educate her family and called her friends on the phone to stay home if necessary and observe protocols at gatherings. In addition, the COVID-19 pandemic has compelled most people to stay home instead of going to the hospital for a checkup. She complained that:

*The living conditions of many older adults in this community are not encouraging. But from what is circulating in the media, I am convinced that the COVID-19 virus is not a joke. I have not come across any COVID-19 patients yet. This raises doubts. Moreover, my children have been telling me about the cases recorded every day (69-year-old-woman)*.

She further states that:

*I would say that may be COVID-19 is attempting to achieve something in the world that we are not aware of. The world system is extremely competitive, and this could lead to nations competing silently. It came, like World War II, and it is likely to determine the next world's Superpower for us in Africa, COVID-19 may be another kind of colonization. From where I sit, even if a vaccine is developed and distributed globally, not everybody will be able to get it (69-year-old-woman)*.

Participant eight, a 71-year-old man, revealed that COVID-19 was created in the laboratory. The main purpose is to wipe out half of the world's population to pave the way for a few individuals to use technology to control humanity. He indicated that staying at home and lockdowns are all attributes of the control of humanity and it tells us that the world has advanced.

*My son, who is a pastor, advised me to pray. His reasoning was that the virus was created in the laboratory to control humanity's everyday life and the global economy in order to push wealth to a few individuals. Any event in our lives, I believe, has a spiritual connotation, and COVID-19 is no exception. I understand that scientists are working hard to find vaccines, but only prayer can save this world and its people (71-year-old-man)*.

### Older Adults' Relationships With Family, Friends, and the Community

Many of the participants explained that, because of COVID-19, their relationships with their families, which serve as a social support unit, have been negatively impacted. Few participants express their view that they have been separated from their social ties and have left them with unsurmountable challenges.

*I live with my family. I have been told to stay home, and I am not happy. Whenever I am at work, I play “Oware (a popular game in Ghana)” with my friends and my apprentice. I have time to play multiple games, and it helps me to think. At home, my children and my wife do not know how to play such games. They only prefer to watch TV and my children usually play games on their phones (67-year-old-man)*.

Furthermore, the study found that many older women were widowed. Three of these women also talked about getting insomnia at night because they sleep alone. They described how not having a husband runs them down in their daily activities. They concluded that, since they do not have their partners around, they find it challenging in the COVID-19 era. One participant expressed her sentiments and lamented during interviews:

*Somebody died of COVID-19, and it scared me to stay indoors. Since that experience, I have not been able to step out of my house. I only watch television, eat, and sleep*. I have children, but the absence of my husband makes it difficult for me. *My social life was thwarted even before COVID-19, but the lockdown has exacerbated it. Although I can go out now, I am still terrified. I feel bad because I had the chance to get married again 10 years ago, but I did not. I become happy when I church. Because the majority of my friends are in church. But church programs have been curtailed because of COVID-19, and we, the older adults, have suffered severely (66-year-old woman)*.

Another participant commented on the fact that she was secure at home, but she was not happy. She described:

*Last year, around March, my brother came to take me to his house. I felt safe. However, I could not attend church and pray. The only thing I did was to join him and his children in the morning for exercises. During the lockdown, I recall everyone was indoors. I have gotten used to sitting in front of this house and receiving greetings from people passing by. The COVID-19 pandemic, on the other hand, has made it impossible to maintain that lifestyle. I have not been able to sit outside since I returned from my brother's house. I am always at home (in-house) with my cousin. I have been getting calls from friends and family members since I returned from my brother's house. The calls have been beneficial and inspiring, but they cannot replace face-to-face and physical interaction with people. I am hoping that COVID-19 will pass to resume my normal social interactions (70-year-old-woman)*.

One male participant complained grievously:

*My friends and I met every Sunday to play “Oware” and “Dame” before COVID-19. It helps us to constantly navigate through our struggles to keep up. Although I have a family, meeting with my childhood friends is something I am missing during this pandemic. What I do now is a conference call with some of my friends, which is not the same as meeting them face-to-face. One of my friends recently died and I could not attend due to COVID-19 procedures (60-year-old-man)*.

Some participants, however, revealed that they had engaged in phone conversations with family members and friends. Frankly speaking, the participants suggested that the phone call experience cannot be compared to their usual engagement with their families and people outside. Participant five revealed that:

*The practice of making phone calls has become the norm. Although I am not a regular person when it comes to phone calls, I have been calling friends and family members to check up on them. They say it is the “New Normal” and I do not have an option anymore. Previously, I used to attend veteran association meetings and from there we would exercise. But reaching out to people over the internet and phone calls is not the same as engaging them in a face-to-face conversation. For the phone calls, I simply call to find out how they are doing (64-year-old-man)*.

### Limited Socialization

The study discovered that older adults' movement and socialization with friends and neighbors has been curtailed by the loose social networks associated with COVID-19. This was partly because COVID-19 non-pharmaceutical protocols have disrupted social gatherings and reduced human movements. As the older adults indicate in their stories, social distancing, reduced face-to-face physical contact and staying at home have broken their social ties and restricted their movement. Almost all the participants have admitted that their inability to socialize with people during the ongoing COVID-19 pandemic event is quite disturbing. In addition, many older adults are isolated because of the fear of getting infected.

*Life without interaction is stressful and traumatic. This pandemic event was accidental. Since the pandemic outbreak, I have been watching television, but television alone cannot account for my happiness and satisfaction. I need to go out and mingle with friends. Sometimes, walking in the sun alone is heartwarming. I believe that, in light of the current COVID-19 situation, people at my age may be isolated and feel helpless (73-year-old-man)*.

Another participant indicated that:

*The movement has become difficult these days. It makes life unbearable. My Social Security and National Insurance Trust (SSNIT) payments are arriving late these days. I have not even received one in over 5 months. COVID-19, I believe, is to be blamed. Ghana is a poor country, and the pandemic has halted a lot of things. Many pensioners were not paying well before COVID-19, and the majority of people are always complaining. The government is confused about the SNNIT funds (70-year-old-woman)*.

### Stress and Depression

Many of the participants reported that they were stressed and depressed because of fear of COVID-19.

*I recall that at the beginning of the pandemic, scientists and researchers told us that it was not an airborne disease. Now they are saying it is airborne. The inconsistency of the pandemic is stressing me (60-year-old-man)*. Another participant, a 69-year-old woman, stated that she had difficulty sleeping. Stress was mentioned several times to demonstrate how the participants were coping with daily life amidst COVID-19. The in-depth interviews revealed how stress left many older adults thinking about how to cope with COVID-19 events.

*My children are taking care of me. They are business people, and their income depends on what they buy and sell. One of them called to inform me that things are very hard for him and his immediate family. What should I eat? she asked. My sons are not meeting their domestic responsibilities and it is headache for me because I depend on them. For me, I have been watching movies to forget about COVID-19 (69-year-old-woman)*.

She further indicated that:

*Recently, my friend passed away and I am not sure if it was the COVID-19 or anything else. Her family called to inform me of her demise. She has a special place in my heart. COVID-19 prevented me from attending the funeral. Sometimes I miss her because she was my childhood friend. We all lived on the same compound. e. Right now, I am not happy as compared to when there was no COVID-19. Ebola scared us. It was only in Africa. But this COVID-19 has placed lives in a total mess, and I can clearly tell that it will take about 5 years for the world to recover from this pandemic (69-year-old-woman)*.

COVID-19-related stress is pervasive among older adults, according to all participants. It is a serious and frequent challenge for them. For instance, participants made it clear how the pandemic has threatened their social engagement with friends and the burden of staying at home for several weeks. Participants revealed that the impossibility of re-uniting and making up with friends and loved ones worries them. Another participant indicated:

*During the lockdown, I felt depressed. The transition was very severe, and I could not make sense of anything. I am self-employed and I have been working on my own since the age of twenty. My family is reliant on me, and although my sons are grown, they are still students. I was fortunate that the children were at home and not paying school fees when COVID-19 halted our movement. I give them money every month if they are in school. Life has become contemptuous to live (62-year-old-man)*.

A 72-year-old male participant revealed his fear of being left out when the vaccine arrived in Ghana. This has put me in a state of worry, and it seems there are only reports about COVID-19 but less support for people like us.

*From what I have heard, there is going to be a vaccine very soon. My concern is how the vaccine can be administered to older adults like myself, who cannot walk or struggle to walk. My inability to go out threatens my life. As you can see, I am unable even to visit the doctor for a checkup. Due to COVID-19, hospitals nowadays do not pay attention to patients properly. Doctors and nurses are afraid of losing their lives—(unless you are severely sick 72-year-old-man)*.

Another participant commented that:

*I am afraid, but there is no one to comfort me. Sometimes I talk to one of my children abroad, but it is just a phone call. It is not the same as having a face-to-face conversation with him. I used to visit my former co-worker, who is very weak and lives alone, but COVID-19 has made it very difficult to reach him. He has a phone, but anytime I call him, it is switched off. I am not sure whether he is still alive. When I go there, we talk about our warm days, laugh a lot, and have a good time. When he was not weak, we drove around and visited places to entertain ourselves (64-year-old-man)*.

### Loneliness

Twelve of the participants expressed concerns about loneliness. Eight of the participants stated that they were able to have conversations with others, but this could not be compared to their lives prior to COVID-19. Participant 6, a 67-year-old woman, expressed frustration that she had no children and was always lonely.

*Previously, before COVID-19, I used to walk whenever I was lonely. I cannot do that anymore because I am terrified of getting infected with this virus. I pray the Lord keeps me forever till he comes, because I always think about him. The COVID-19 pandemic has worsened my case. I have stopped watching television because they do not give any encouraging information about this pandemic.—(67-year-old woman)*.

## Discussion

In this study, we have documented the experiences of older adults, as well as the impact of the COVID-19 pandemic on their social support networks, which has disrupted many people's lives. Participants who had been exposed to COVID-19 but were not infected at the time of the investigation provided the perspectives reflected in this study. The outcomes of the findings indicated that participants draw on the following themes connected to older adult opinions on the impact of COVID-19: perception about COVID-19, older adults' relationships with social networks (family, friends, community), limited socialization, stress, depression, and loneliness. Participants specifically stated that the COVID-19 preventative measures like stay-at-home and lockdown, which included limited movement, increased loneliness, and stress among older adults. Even though participants described their social networks as diverse and helpful, the psychological distress associated with the COVID-19 pandemic has wreaked havoc on their relationships. Primarily, the findings demonstrated, first and foremost, that the benefits of social networks influenced the outcome of older adults' wellbeing, which had previously relied on family systems and community assistance had fallen short of expectations during COVID-19.

Participants highlighted several changes that had affected their networks and care systems. COVID-19 precautionary measures, such as social distancing, and reducing physical and face-to-face interactions with friends and family members, for example, have been reported to disrupt relationships among older adults and their social networks, confirming an increase in social isolation (34, 35). For example, it has been established that being in regular contact with social networks promotes life satisfaction and reduces depression and isolation in older adults (36). However, the fear of getting infected with or passing the virus to others exacerbated the lack of support for older adults from family and friends and the healthcare system.

The study found that the effects of COVID-19 on participants' social support networks has resulted in widespread stress, sadness, and loneliness among older persons, which was predicted by multiple previous studies (37–39). On the other hand, some studies have indicated that social isolation caused by non-pharmaceutical pandemic measures did not result in increase in loneliness (40, 41). Nonetheless, majority of the participants expressed dissatisfaction with the COVID-19 precautionary measures, claiming that they have been cut off from their families, friends, and neighbors. It is important to add that few participants reported using their cellphones to contact friends and family. Watching television verified the use of digital technology as a new method for older folks to maintain frequent social ties (26). Evidence suggests social networks plays an important role in assisting individuals, including older adults, during natural catastrophes and pandemics (42). However, the study revealed that COVID-19 has rendered the social support networks of older adults ineffective, since many older persons and their families are financially stressed, their children are jobless, and they have poor living conditions. Therefore, these findings may further be linked to socio-economic disadvantaged (43), which causes majority of vulnerable people to be impoverished, as well as poor distribution of pension income by the Social Security and National Insurance Trust [SSNIT] to retirees in Ghana (44).

Another major theme in this study was the loneliness that resulted from the COVID-19, particularly during the lockdown period. The loneliness experienced by older persons in this study confirms pervious report by the World Health Organization's (2) argument that the disruption of social networks linked to COVID-19 may negatively affect older adults' mental health and psychological well-being (45). The deleterious effects of COVID-19 on older adults are discovered to be exacerbated by lockdown, causing psychological problems (43, 46). Loneliness has been reported as one of the COVID-19 prevalence consequences, ensuing disruption of social networks (42, 47–50). These scholars have emphasized the necessity of equipping family members and relatives and residential care homes to assist the elderly in their care. It is commonly established that the support and wellness of older adults, particularly older persons with strong networks, will work more efficiently when viewed through the lens of social networks. The relevance of engaging in social networks for the mental health and wellbeing of older adults give them a sense of belonging and remove stigmatization during pandemics.

The findings suggest that participants experienced stress and depression as a result of fear and anxiety. The fear of getting infected and the misinformation about the COVID-19 pandemic created tension among people across the world. This situation has rendered most older adults isolated and stigmatized since they have limited access to perform their daily activities and less interaction with family members, friends, and associations. This backs up a slew of evidence of isolation and stigmatization issues that have been highlighted in several COVID-19 research and reports (51, 52). As a result, older adults are more likely to experience social isolation and loneliness. Members of a traditional African community, such as Jamestown, are identified and associated with one another, allowing them to socialize without fear, whereas COVID-19 has made it difficult to do so. This indicates that there is a need for the government to strengthen its policies and pay critical attention to the rights of older adults.

Even though participants were severely affected by the COVID-19 pandemic, findings revealed that some older adults in Jamestown remain skeptical about the pandemic's reality. They believed the virus, which is found to be airborne, could not survive in a salty environment. In addition, participants indicated that many deaths had been reported in Jamestown community, which they believed were unrelated to COVID-19. But the fact that some participants agree that no one had been infected or died from the virus was a high point in this study.

For scholars working in the COVID-19 literature, particularly in Ghana, this study is significant from a methodological standpoint. COVID-19 has been the subject of several investigations (23–26, 53), but this study primarily reports the voices and perspectives of older adults in Jamestown community. Previous studies on the impact of COVID-19 on older persons in Ghana was based on analysis of the state of older adults' social support networks preceding up to COVID-19 (25, 48), this empirical study evaluates information gathered through interviews. This appears to be a bold step in the direction of conveying the story of those who are susceptible to the COVID-19 pandemic. However, the present study's sample size was hampered by COVID-19 restrictions, which have affected face-to-face physical contact interactions. Wu and McGoogan (34) reported that the increased mortality rate reflects the aging population's inherent biological, social, and psychological vulnerabilities. While the study points to a growing fear of COVID-19 among older adults, chronic diseases still pose a significant threat to the health and wellbeing of many older adults. The prevalence of fear and depression accompanied by COVID-19 as documented in this study should be a concern for social workers, advocates, and the government to improve the work of social workers such that family ties, friends, and neighbors can offer long-term assistance to older adults in Ghana (48). Policymakers should consider allocating greater resources to caregivers and institutional care interventions, especially for older adults who are living in vulnerable communities with poor conditions and without proper accommodation.

Morgan and Awafo (54) reported that the recent outbreak of the COVID-19 pandemic has imposed a heavy burden on individuals' wellbeing, the world economies, financial, healthcare systems, and lifestyles, which are under distinct threats. The data available indicates that more than 95% of COVID-19 deaths are found among older adults over 60 years of age, and half of all deaths occur at 80 years-plus (2). Early reports suggested that older adults with chronic diseases like diabetes 2 and high blood pressure are more vulnerable to coronavirus disease due to their weak immune systems (2). Research has shown that an estimated 66% of people aged 60 years and over, at least, have one underlying condition, placing them at a higher risk of COVID-19 (55). Furthermore, depression, suicide, stress and substantial mental health issues are all outcomes connected to COVID-19, as well as discrimination, stigma and the disruption of social networks (55). Furthermore, this study, done in Jamestown-Accra, cannot represent widespread opinion due to variances in perceptions of the COVID-19 pandemic and inequitable living conditions among Ghana's older adults. As a result, more research into this phenomenon is required to portray the whole picture of COVID-19 and social networks among Ghana's older individuals.

Nonetheless, this study adds to our knowledge of the state of social networks among older adults in the face of pandemics in the 21st century. The study's strength lies in the fact that it analyzes and interprets data derived from empirical data. Nonetheless, the study's conclusions are limited because they cannot be applied to Ghana's broader population of older adults. Although COVID-19 cases were confirmed in Jamestown, the experiences and problems may not have been the same. These findings show that efforts to advocate for older individuals' rights and a collaborative and integrative approach to helping them should not be overlooked.

## Conclusions

Covid-19 has disrupted older adult's long-lasting social networks resulting in mental and psychological issues such as worry about finances and wellbeing among older adults worldwide. This study has revealed that there is a pressing need to reconsider the professional services of gerontologists, social workers, community outreach workers, and philanthropists in mitigating loneliness among older adults in future pandemics. Strong activism is required to assure the passage of an aging bill that will improve the lives of older adults in the event of future pandemics. Moreover, the project featuring technological advancement and accessibility should be embarked to help older adults social through social media even in lockdowns.

## Data Availability Statement

The original contributions presented in the study are included in the article/supplementary material, further inquiries can be directed to the corresponding authors.

## Ethics Statement

The studies involving human participants were reviewed and approved by the study was proceeded by acquiring a formal ethical clearance certificate (ref: CHRPE/AP/108/21) from the Committee on Human Research, Publication, and Ethics (CHRPE) of the Kwame Nkrumah University of Science and Technology, Kumasi. The patients/participants provided their written informed consent to participate in this study.

## Author Contributions

All authors listed have made a substantial, direct and intellectual contribution to the work, and approved it for publication.

## Conflict of Interest

The authors declare that the research was conducted in the absence of any commercial or financial relationships that could be construed as a potential conflict of interest.

## Publisher's Note

All claims expressed in this article are solely those of the authors and do not necessarily represent those of their affiliated organizations, or those of the publisher, the editors and the reviewers. Any product that may be evaluated in this article, or claim that may be made by its manufacturer, is not guaranteed or endorsed by the publisher.

## References

[1] Kaniasty K. Predicting social psychological well-being following trauma: The role of post disaster social support. Psychological Trauma: Theory, Research, Practice, and Policy. (2012) 4:22. 10.1037/a00214122624130

[2] World Health Organisation. (2020). Older adults and Covid-19. Retrieved on 2 April, 2021, from https://www.who.int/teams/social-determinants-of-health/covid-19/ (accessed September 10, 2021).

[3] Pérez-Escoda A Jiménez-Narros C Perlado-Lamo-de-Espinosa M Pedrero-Esteban LM. Social networks' engagement during the COVID-19 pandemic in Spain: health media vs. healthcare professionals. Int J Envir Res Public Health. (2020) 17:5261. 10.3390/ijerph17145261 32708231 PMC7400399

[4] Killgore WD Cloonan SA Taylor EC Dailey NS. Loneliness: A signature mental health concern in the era of COVID-19. Psychiatry Res. (2020) 290:113117. 10.1016/j.psychres.2020.113117 32480121 PMC7255345

[5] Kovacs B Caplan N Grob S King M. Social networks and loneliness during the COVID-19 pandemic. Socius. (2021) 7:2378023120985254. 10.1177/2378023120985254

[6] Antonucci TC. Social relations: An examination of social networks, social support, and sense of control. In: Birren JE, Editors. Handbook of the psychology of aging (5th ed.). San Diego, CA: Academic Press. (2001). pp. 427–53

[7] Chi I Chou KL. Social support and depression among Hong Kong Chinese older adults. Int J Aging Hum Dev. (2001) 52:231–51. 10.2190/V5K8-CNMG-G2UP-37QV 11407488

[8] Yuan C. W., Kropczynski, J., Wirth, R., Rosson, M. B., and Carroll, J. M. Investigating older adults' social networks and coproduction activities for health. In: *Proceedings of the 11th EAI International Conference on Pervasive Computing Technologies for Healthcare*. (2017). p. 68–77. 10.1145/3154862.3154876

[9] Litwin H Landau R. Social network type and social support among the old-old. J Aging Stud. (2000) 14:213–28. 10.1016/S0890-4065(00)80012-223540157

[10] Onder G Rezza G Brusaferro S. Case-fatality rate and characteristics of patients dying in relation to COVID-19 in Italy. JAMA. (2020) 323:1775–6. 10.1001/jama.2020.4683 32203977

[11] Sanyaolu A Okorie C Marinkovic A Patidar R Younis K Desai P. Comorbidity and its Impact on Patients with COVID-19. SN Comprehensive Clinical Medicine. (2020) 1–8. 10.1007/s42399-020-00363-4 PMC7314621 32838147

[12] Zhou F Yu T Du R Fan G Liu Y Liu Z. Clinical course and risk factors for mortality of adult inpatients with COVID-19 in Wuhan, China: a retrospective cohort study. Lancet. (2020) 395:1054–62. 10.1016/S0140-6736(20)30566-3 32171076 PMC7270627

[13] Adjei P Afriyie-Mensah J Ganu VJ Puplampu P Opoku-Asare B Dzefi-Tettey K. Clinical characteristics of COVID-19 patients admitted at the Korle-Bu Teaching Hospital, Accra, Ghana. Ghana Medical J. (2020) 54:33–8. 10.4314/gmj.v54i4s.6 33976439 PMC8087357

[14] Yawson AE Oduro-Mensah E Tetteh J Adomako I Adjei-Mensah E Owoo C. Clinical features of COVID-19 in Ghana: symptomatology, illness severity and comorbid non-communicable diseases. Ghana Med J. (2020) 54:23–32. 10.4314/gmj.v54i4s.5 33976438 PMC8087366

[15] Awuviry-Newton K Wales K Tavener M Byles J. Do factors across the World Health Organisation's International Classification of Functioning, Disability and Health framework relate to caregiver availability for community-dwelling older adults in Ghana? PLoS ONE. (2020) 15:e0233541. 10.1371/journal.pone.0233541 32469915 PMC7259767

[16] Payne CF Mkandawire J Kohler HP. Disability transitions and health expectancies among adults 45 years and older in Malawi: a cohort-based model. PLoS Med. (2013) 10:e1001435. 10.1371/journal.pmed.1001435 23667343 PMC3646719

[17] Biritwum RB Minicuci N Yawson AE Theou O Mensah GP Naidoo N et al. (2016). Prevalence of and factors associated with frailty and disability in older adults from China, Ghana, India, Mexico, Russia and South Africa. Maturitas. 91:8–18. 10.1016/j.maturitas.2016.05.012 27451316

[18] Litwin H. The network shifts of elderly immigrants: The case of Soviet Jews in Israel. J Cross Cult Gerontol. (1997) 12:45–60. 10.1023/A:1006593025061 14617939

[19] Nukunya GK. Tradition and Change in Ghana: An Introduction to Sociology. Ghana Universities Press (2003).

[20] Schaefer C Coyne JC Lazarus RS. The health-related functions of social support. J Behav Med. (1981) 4:381–406. 10.1007/BF00846149 7338894

[21] Gupta M Abdelmaksoud A Jafferany M Lotti T Sadoughifar R Goldust M. COVID-19 and economy. Dermatol Ther. (2020). 10.1111/dth.13329 PMC7228404 32216130

[22] Kanu IA. COVID-19 and the economy: an African perspective. J African Studies and Sustainable Development. (2020) 3. 10.13140/RG.2.2.18801.43362

[23] Yezli S Khan A. COVID-19 social distancing in the Kingdom of Saudi Arabia: Bold measures in the face of political, economic, social and religious challenges. Travel Med Infect Dis. (2020) 37:101692. 10.1016/j.tmaid.2020.101692 32330561 PMC7172679

[24] Asiamah N Opuni FF Mends-Brew E Mensah SW Mensah HK Quansah F. Short-Term changes in behaviors resulting from COVID-19-Related social isolation and their influences on mental health in Ghana. Community Ment Health J. (2021) 57:79–92. 10.1007/s10597-020-00722-4 33033971 PMC7543965

[25] Deku CS Forkuor JB Agyemang E. COVID 19 meets changing traditional care systems for the elderly and a budding social work practice. Reflections for geriatric care in Ghana. Qual Soc Work. (2021) 20:501–6. 10.1177/1473325020973323 34253991 PMC8261343

[26] Ahiadorme A Esenam. The Untold Story of Older adults in Ghana. Retrieved on 10 January, 2021. (2018) Available online at: https://www.thepublisheronline.com/the-untold-story-of-older-people-in-ghana/ (accessed October 20, 2021).

[27] Langdridge D. Phenomenological psychology: theory, research and method. Pearson Education Ltd. (2007).

[28] Awuviry-Newton K Nkansah JO Ofori-Dua K. Attributions of elder neglect: A phenomenological study of older adults in Ghana. Health Soc Care Community. (2020) 28:2172–8. 10.1111/hsc.13028 32378297

[29] Cresswell JW. Qualitative Inquiry. Thousands Oak, CA (2007).

[30] Ghana Statistical Service. (2013). Population and housing census report. Available online at: www.statsghana.gov.gh/docfiles/2010phc/National_Analytical_Report.pdf.

[31] Braun V Clarke V. Using thematic analysis in psychology. Qual Res Psychol. (2006) 3:77–101. 10.1191/1478088706qp063oa32100154

[32] Kiger ME Varpio L. Thematic analysis of qualitative data: AMEE Guide No. 131. Medical Teacher. (2020) 42:846–54. 10.1080/0142159X.2020.1755030 32356468

[33] Ansah EW Sarfo JO Apaak D. Physical activity and dietary behaviors: a phenomenological analysis of experiences of Ghanaians during the COVID-19 lockdown. Pan Afr Med J. (2020) 37:199. 10.11604/pamj.2020.37.199.23733 33505568 PMC7813653

[34] Wu Z McGoogan JM. Characteristics of and important lessons from the coronavirus disease 2019 (COVID-19) outbreak in China: summary of a report of 72 314 cases from the Chinese Center for Disease Control and Prevention. JAMA. (2020) 323:1239–42. 10.1001/jama.2020.2648 32091533

[35] Malik M Burhanullah H Lyketsos CG. Elder abuse and ageism during COVID-19. Psychiatric Times (2020).

[36] Chan YK Lee RP. Network size, social support and happiness in later life: A comparative study of Beijing and Hong Kong. J Happiness Stud. (2006) 7:87–112. 10.1007/s10902-005-1915-1

[37] Armitage R Nellums LB. COVID-19 and the consequences of isolating the elderly. The Lancet Public Health. (2020) 5:e256. 10.1016/S2468-2667(20)30061-X 32199471 PMC7104160

[38] Berg-Weger M., and Morley, J. E. Loneliness and social isolation in older adults during the Covid-19 pandemic: Implications for gerontological social work. J Nutrition, Health Aging. (2020) 24:456–458. 10.1007/s12603-020-1366-8 32346678 PMC7156792

[39] Smith BJ Lim MH. How the COVID-19 pandemic is focusing attention on loneliness and social isolation. Public Health Research & Practice. (2020) 30:3022008. 10.17061/phrp3022008 32601651

[40] Benke C., Autenrieth, L. K., Asselmann, E., and Pané-Farr,é, C. A. Stay-at-home orders due to the COVID-19 pandemic are associated with elevated depression and anxiety in younger, but not older adults: results from a nationwide community sample of adults from Germany. Psychological Medicine. (2020) 1–2. 10.1017/S0033291720003438 PMC7487742 32895064

[41] Kotwal AA Holt-Lunstad J Newmark RL Cenzer I Smith AK Covinsky KE. Social isolation and loneliness among San Francisco Bay Area older adults during the COVID-19 shelter-in-place orders. J Am Geriatr Soc. (2021) 69:20–9. 10.1111/jgs.16865 32965024 PMC7536935

[42] Saltzman LY Hansel TC Bordnick PS. Loneliness, isolation, and social support factors in post-COVID-19 mental health. Psychological Trauma: Theory, Research, Practice, and Policy. (2020) 39:S57. 10.1037/tra0000703 32551762

[43] Osimo SA Aiello M Gentili C Ionta S Cecchetto C. The influence of personality, resilience, and alexithymia on mental health during COVID-19 pandemic. Front Psychol. (2021) 12:341. 10.3389/fpsyg.2021.630751 33716896 PMC7943855

[44] Nkansah OJ Awuviry-Newton K Gyasi M Newton A Boateng ASA. Who doesn't Have Challenges? I Have a Lot of Challenges: Exploring the Challenges and Coping Strategies of Neglected Older Adults in Ghana. Journal of Cross-Cultural Gerontology. (2021) 1–14. 10.1007/s10823-020-09419-3 33400080

[45] World Health Organisation. Older People and Covid-19 (2020). Available online at: https://www.who.int/teams/social-determinants-of-health/covid-19/

[46] Cecchetto C Aiello M Gentili C Ionta S Osimo SA. Increased emotional eating during COVID-19 associated with lockdown, psychological and social distress. Appetite. (2021) 160:105122. 10.1016/j.appet.2021.105122 33453336 PMC9755826

[47] Alheneidi H AlSumait L AlSumait D Smith AP. Loneliness and Problematic Internet Use during COVID-19 Lock-Down. Behavioral Sci. (2021) 11:5. 10.3390/bs11010005 33418914 PMC7825032

[48] Arthur-Holmes F Agyemang-Duah W. Reaching older adults during the COVID-19 pandemic through social networks and Social Security Schemes in Ghana: Lessons for considerations. J Gerontol Soc Work. (2020) 63:699–701. 10.1080/01634372.2020.1764689 32449640

[49] Groarke JM Berry E Graham-Wisener L McKenna-Plumley PE McGlinchey E Armour C. Loneliness in the UK during the COVID-19 pandemic: Cross-sectional results from the COVID-19 Psychological Wellbeing Study. PLoS ONE. (2020) 15:e0239698. 10.1371/journal.pone.0239698 32970764 PMC7513993

[50] Schellekens MP van der Lee ML. Loneliness and belonging: Exploring experiences with the COVID-19 pandemic in psycho-oncology. Psychooncology. (2020) 29:1399–401. 10.1002/pon.5459 32628307 PMC7361721

[51] Hwang TJ Rabheru K Peisah C Reichman W Ikeda M. Loneliness and social isolation during the COVID-19 pandemic. International Psychogeriatrics. (2020) 32:1217–20. 10.1017/S1041610220000988 32450943 PMC7306546

[52] Kwaghe AV Ilesanmi OS Amede PO Okediran JO Utulu R Balogun MS. Stigmatization, psychological and emotional trauma among frontline health care workers treated for COVID-19 in Lagos State, Nigeria: A qualitative study. BMC Health Serv Res. (2021) 21:1–13. 10.1186/s12913-021-06835-0 34419034 PMC8380097

[53] Pelicioni PH Lord SR. *COVID-19 will severely impact older adults's* lives, and in many more ways than you think! Brazilian journal of physical therapy. (2020) 10.1016/j.bjpt.2020.04.005 PMC7252007 32387005

[54] Morgan AK Awafo BA. Lessons for Averting the Delayed and Reduced Patronage of non-COVID-19 Medical Services by Older adults in Ghana. J Gerontol Soc Work. (2020) 63:728–31. 10.1080/01634372.2020.1808142 32807031

[55] United Nations. (2020). Policy Brief: The impacts of covid-19 on older persons. Available online at: https://www.un.org/sites/un2.un.org/files/un_policy_brief_on_covid-19_and_older_persons_1_may_2020.pdf (accessed September 15, 2021).

